# A model of interprofessional problem-based learning for medical and nursing students: Implementation, evaluation and implications for future implementation

**DOI:** 10.3205/zma001160

**Published:** 2018-02-15

**Authors:** Adina Dreier-Wolfgramm, Sabine Homeyer, Roman F. Oppermann, Wolfgang Hoffmann

**Affiliations:** 1 University Medicine Greifswald, Institute for Community Medicine, Section Epidemiology of Health Care and Community Health, Greifswald, Germany; 2 University of Applied Sciences Neubrandenburg, Department of Health, Nursing and Management, Neubrandenburg, Germany

**Keywords:** interprofessional education, problem-based learning, interdisciplinary studies, evaluation research

## Abstract

**Objective: **In Germany there is little evidence of interprofessional problem-based learning (PBL) to date. For this reason a corresponding course for medical and nursing students was implemented. The goal was to analyse the feasibility and usefulness from the point of view of the students.

**Method: **For the purposes of evaluation a quantitative observational study was conducted with a pre-course survey, a teaching evaluation and a post-course survey. The pre- and post-course surveys took place before the commencement of and after the conclusion of learning. The teaching evaluation was carried out after the conclusion of the interprofessional course. In total there were five medical students and five nursing students who took part. The descriptive data analysis was carried out using the statistics software package SPSS. The data from the open text fields was transcribed and evaluated using qualitative content analysis.

**Results: **The students’ assessment of the interprofessional PBL was predominantly positive. They stated that they

1. were able to expand their knowledge of the roles of the other profession, that
2. the interprofessional course had a positive effect in terms of mutual appreciation and that
3. the tutor had a positive influence on the interprofessional PBL due to his cooperative learning support.

Suggestions for improvement were concerned with the expansion of the interprofessional exchange and the establishment of a set timetable for the tutorials.

**Conclusion: **The first study results of the Bildungscluster study can be used to make adjustments to the interprofessional PBL in order to be able to implement the course with a greater number of participants. Further studies are needed in order to comprehensively examine the learning effects and the impact on daily practice.

## Zusammenfassung

**Zielsetzung: **In Deutschland besteht bislang wenig Evidenz zum interprofessionellen problembasierten Lernen (PBL). Daher erfolgte die Implementierung einer entsprechenden Lehrveranstaltung für Medizin- und Pflegestudierende. Ziel war es, die Machbarkeit und den Nutzen aus Sicht der Studierenden zu analysieren.

**Methodik: **Zur Evaluation erfolgte eine quantitative Beobachtungsstudie mit einer Pre-Befragung, Lehrevaluation und einer Post-Befragung. Die Pre-Post Befragung erfolgte vor Beginn und nach Abschluss des Lernens. Die Lehrevaluation wurde nach Abschluss der interprofessionellen Lehrveranstaltung durchgeführt. Insgesamt haben fünf Medizinstudierende und fünf Pflegestudierende teilgenommen. Die deskriptive Datenanalyse erfolgte mit der Statistiksoftware SPSS. Die Daten der Freitextfelder wurden transkribiert und nach der qualitativen Inhaltsanalyse ausgewertet.

**Ergebnisse: **Die Studierenden haben das interprofessionelle PBL überwiegend positiv bewertet. Sie geben an, dass sie

1. ihre Kenntnisse zu den Rollen der anderen Profession erweitern konnten, dass sich
2. die interprofessionelle Lehrveranstaltung positiv auf die gegenseitige Wertschätzung ausgewirkt hat und dass
3. der Tutor durch seine kooperative Lernbegleitung das interprofessionelle PBL positiv beeinflusst hat.

Verbesserungsvorschläge betreffen die Ausweitung des interprofessionellen Austausches und die Festlegung von festen Terminen für die Tutorien.

**Schlussfolgerung:** Die ersten Studienergebnisse der Bildungsclusterstudie können zur Anpassung des interprofessionellen PBLs genutzt werden, um die Lehrveranstaltung mit einer größeren Anzahl an Teilnehmern umsetzen zu können. Weitere Studien sind erforderlich, um die Lerneffekte und Auswirkungen auf den Praxisalltag umfassend zu untersuchen.

## 1. Introduction

### 1.1. Interprofessional problem-based learning

The increasing workload and the growing complexity of the treatment of patients will in future require increased collaboration between the healthcare professions. Improved interprofessional teamwork constitutes one of the main expected effects of interprofessional education (IPE) [1]. Studies have shown that good collaboration between the healthcare professions contributes to improvements in terms of patient safety and to a reduction in treatment errors [2], [3], [4]. The World Health Organisation (WHO) thus already identified the need for IPE in 1988 [5]. From the point of view of the WHO, healthcare professions’ knowledge of their own roles and the roles of the other health care professionals are one of the indispensable competencies for good interprofessional work within a team. The development of this kind of understanding of roles can be trained extensively within the scope of problem-based learning (PBL). Consequently, PBL represents a suitable learning approach for IPE [6].

In 2010 Thompson et al. reviewed the literature about the effects of the combination of IPE and PBL and came to the conclusion that interprofessional PBL improved the attitudes of the health care professions towards each other [7]. Cusack et al. and Imafuku et al. add that IPE can have a positive influence on mutual respect and trust [4], [8]. These are important prerequisites in order to provide healthcare professions with motivation for interprofessional collaboration and to adequately prepare them for it within the framework of interprofessional PBL [4], [9], [10]. Specifically, learning in small groups with an interactive learning approach enables the group members to learn how to work as members of a team and to solve problems as a team [4], [8], [11], [12]. Consequently interprofessional PBL has already been extensively implemented internationally.

#### 1.2. An exemplary implementation of interprofessional problem-oriented learning for nursing and medical students: The Bildungscluster study Greifswald/Neubrandenburg

In Germany to date there is little evidence of the implementation of interprofessional PBL and its effects on the participating health care professional groups. For this reason an interprofessional course using PBL was implemented within the context of the Bildungscluster study Greifswald/Neubrandenburg. The interprofessional PBL took place in “Community Medicine II - Das Hospitationsprogramm” (Community Medicine II – The work shadowing programme). This course is a compulsory part of the regular curriculum for the medical students at University Medicine Greifswald (UMG) and is already conducted with PBL as a method. The integration of interprofessional PBL into an existing course was chosen in order to be able to permanently implement IPE in the future and to simplify the transfer into the regular curriculum of both professional training courses.

**Figure 1:**
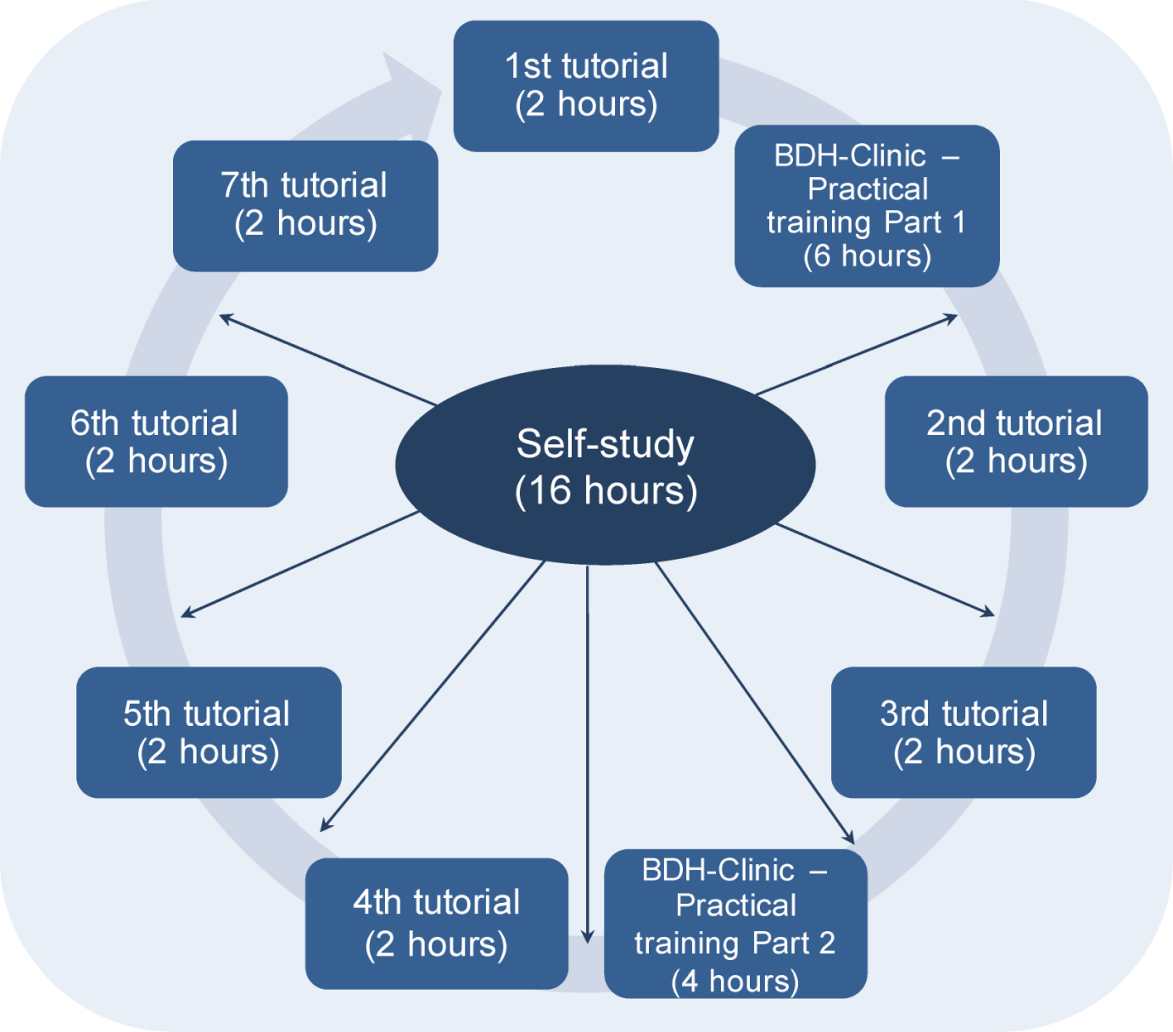
Composition and structure of the interprofessional course

This course comprised a total of 42 academic hours of 45 minutes length with the following format (see Figure 1 (Fig. 1)):

1. Practical training at the BDH-Clinic Greifswald (neurological rehabilitation centre and treatment centre for paraplegics) (10 hours): This part of the course was conducted on two days in the BDH-Clinic. The aim was to look in detail at the facility and its work processes. On the first day of the practical training the participants also became familiar with the patient clientele and received information about the accessibility of the facility for the patients. On the second day of the practical training the participants had the chance to look, by way of example, at a patient in more detail (access to the patient records with the patient history and the course of treatment in the BDH-Clinic) as well as to conduct an assessment about case history with regard to the current treatment situation. Furthermore the students were divided into two-person teams (each with one medical and one nursing student). The patients for the assessment about case history were selected by BDH-Clinic personnel and was carried out by the two-person teams using a medical history form from the BDH-Clinic. Additionally, the participants asked the questions which they themselves had formulated in order to answer the question in their assignment (e.g. What is the patients’ assessment of the current collaboration between doctors and nurses in the hospital? Where do they see room for improvement?, What are the patients’ wishes in terms of collaboration?, etc.). The questions were independently developed by the participants in the second and third tutorials.
2. Seven tutorials (14 hours): The tutorials took place in the UMG and served to tackle the question which the participants developed in the first tutorial and after the first day of practical training in the BDH-Clinic using the seven-step PBL method. An additional starting point was the written description of the BDH-Clinic facility (history of the facility, common clinical pictures, treatment methods, etc.), which was developed by the Institute for Community Medicine (ICM) of the UMG. The participants dealt with the problem of the shortage of qualified personnel in medicine and nursing with the following question: “Can the improved interprofessional collaboration of nursing and medicine make a contribution towards combatting the skills shortage?”. The goal was to compile the results of the work on the question in a joint assignment.
3. Self-study (16 hours): The self-study occurred between the tutorials and was used by each student individually for the development of the joint assignment. Furthermore, tasks were assigned to each participant during the tutorials.
4. Colloquium (2 hours): In the colloquium the group of participants presented the completed assignment with the questions and results. The tutor, who provided advice and guidance in a cooperative mode in the interprofessional course [13], gave the group feedback about the cooperation during the tutorials. An employee from the ICM announced the assessment of the assignment according to the evaluation criteria.

#### 1.3. Questions and objective

The following questions were examined:

1. What is the students’ evaluation of the course structure and the tutor’s guidance of the group processes?,
2. What are the learning effects? and
3. What further topics are suitable for interprofessional PBL in the eyes of the students?

The goal was to identify the assessment of the feasibility and usefulness of interprofessional PBL from the viewpoint of the students. This is the starting point in order to organise interprofessional PBL as optimally as possible in future for both health care professions and to be able to permanently implement this specific interprofessional teaching format in the regular curriculum of both professions.

## 2. Method

### 2.1. Study design

For the evaluation there was a quantitative observational study which consisted of a pre-course survey, the teaching evaluation and a post-course survey. Details of the study have already been published elsewhere [14], [15]. The pre- and post-course surveys mainly served to describe the students’ mutual appreciation and knowledge of roles. Afterwards, further suitable topics for interprofessional PBL were to be identified. The teaching evaluation was designed to analyse the structure and schedule of the course as well as the learning effects and implications for a lasting implementation.

#### 2.2. Students

In total 10 students (n=5 medical students from University Medicine Greifswald (UMG), n=5 nursing students from the University of Applied Sciences Neubrandenburg (HS NB) took part in the interprofessional PBL. Both groups of students were in the first year of their studies and had no previous experience of IPE. The recruitment took place within the framework of the welcoming event for new students in their first semester. The students were informed about the Bildungscluster study and how it would be run and were also invited to participate. Additionally, they received written information about the study and a consent form. The first five medical and nursing students who presented the written informed consent were included in the Bildungscluster study.

Four medical students were male (average age 26, SD: 4.0). Two medical students had completed a vocational nursing training course prior to commencing their studies. Four nursing students were female (average age 25, SD: 3.0) and had completed vocational nursing training before commencing their nursing studies.

#### 2.3. Instructors

The practical training in the BDH-Clinic was guided by a doctor and a nurse from the BDH-Clinic. They made the participants aware of the patient clientele and the accessibility of the facility for the patients. At the same time both instructors introduced the work processes in the BDH-Clinic and were responsible for the selection of the patients for the asessment about case history.

The guidance during the tutorials took the form of peer-teaching by a student tutor who was a medical student in the clinical part of his training (second clinical year). He had completed training as an emergency paramedic before commencing his studies. This ensured that the tutor was also able to adequately take into account the perspectives of other healthcare professions, in this case that of the nursing students.

#### 2.4. Data collection

When the study began there were no valid and reliable instruments available for the evaluation of interprofessional PBL (see Section 4 Discussion). Thus, within the context of the study, two data collection instruments were developed for the pre- and post-course survey and the teaching evaluation.

The questionnaire for the pre- and post-course survey looked at a total of 11 items covering three dimensions:

1. IPE,
2. the roles of both professions, and also
3. suitable topics for interprofessional PBL.

Nine items were evaluated with a four-step Likert scale. The mutual description of one’s knowledge of the other profession’s role and the naming of suitable topics for interprofessional PBL were completed in open text fields.

The instrument for the teaching evaluation included 13 items in a questionnaire covering three dimensions:

1. The course structure and the tutor (three items),
2. learning effects (seven items) and
3. suggestions for improvement (three items).

Eight items were answered using a four-step Likert scale. The assessment of the group size for a successful learning process was made using the answer categories:

1. just right,
2. too small,
3. too big.

Four items (description of the learning effects (1 item) and suggestions for improvement (three items) were answered in open text fields.

Both data collection instruments were pre-tested with a small group of six students from both professions and subsequently digitalised in TeleForm® (Electric Paper Information Systems GmbH Lüneburg, version 10.2). The students completed the pre- and post-course questionnaire anonymously before the commencement of and after the conclusion of the interprofessional PBL. The teaching evaluation took place after the conclusion of the interprofessional course. The completed questionnaires were scanned and verified in TeleForm®. For the purposes of analysis the data collected was transferred to the IBM® SPSS® Statistics Programme (version 22, Ehningen, Germany).

#### 2.5. Data analysis

The descriptive data analysis was conducted using the SPSS statistics software. The data from the open text fields was transcribed and evaluated using qualitative content analysis according to Kuckartz [16] and the software MAXQDA (version 12, VERBI GmbH, Berlin, Germany). Two members of the study team coded the data according to the principle of consensual coding [16].

## 3. Results

### 3.1. Design of the interprofessional course

Overall, the interprofessional PBL was predominantly evaluated by both groups of students as being either good or satisfactory (see Table 1 (Tab. 1)). It is noteworthy that, compared to the nursing students, the medical students evaluated the design as less successful.

From the point of view of both groups of students it was largely possible to learn the course contents in the given time. The link between theory and practice was only viewed by four students (n=2 nursing, n=2 medical) to have been successful (see Table 1 (Tab. 1)).

Nine students felt that the size of the group was just right for effective learning. Four students (n=2 medical, n=2 nursing) assessed their level of knowledge after the conclusion of the course as “higher”. Three students (n=1 medical, n=2 nursing) indicated that they were barely able to expand their knowledge.

A total of six students (n=3 medical, n=3 nursing) rated the interprofessional PBL as being a “very interesting” or “interesting” course. Five students (n=3 nursing, n=2 medical) would participate in the interprofessional PBL course again in this form. Three students (n=2 medical students, n=1 nursing students) stated that they would be unlikely to participate in the course again in this form.

#### 3.2. Evaluation of the tutor

The tutor’s guidance and support of the group of learners was evaluated predominantly positively by both groups of students (see Table 2 (Tab. 2)). From the viewpoint of the students, the tutor was particularly successful at providing support to the group members when they experienced learning difficulties and also at giving them the motivation to actively participate and exchange ideas with others in the group.

The tutor was successful in doing so, among other things, through the feedback he provided with regard to the contribution made by the individual students (see Table 2 (Tab. 2)).

#### 3.3. Learning effects

##### 3.3.1. Mutual understanding of roles

**Figure 2:**
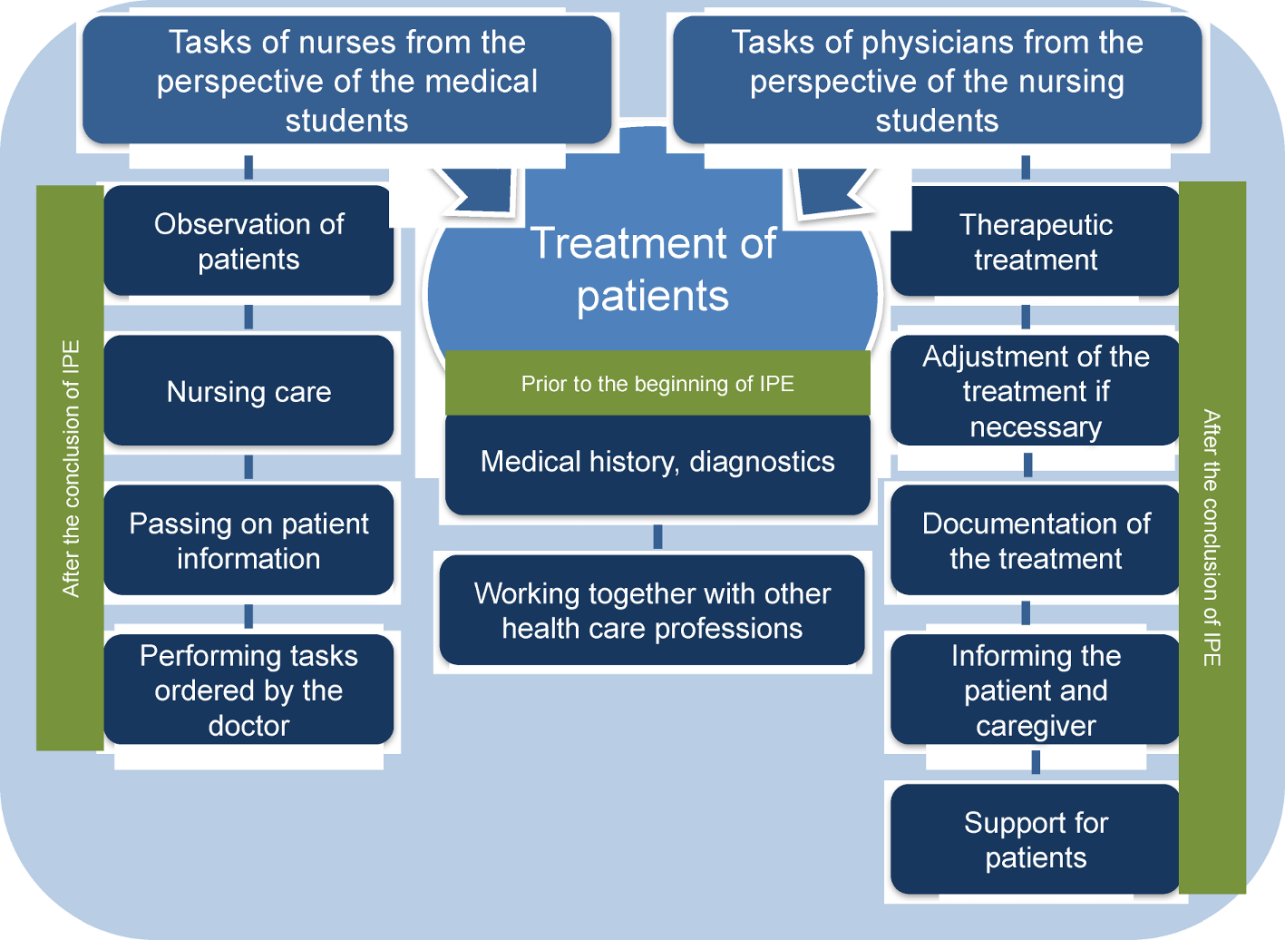
Students’ knowledge of the role of the respective other profession (N=10)

Before the interprofessional PBL began, both groups of students stated with regard to the roles of medicine and nursing that they knew the tasks of the respective other profession well. Participation in the interprofessional course led to the students having an expanded and more detailed understanding of the roles (see Figure 2 (Fig. 2)).

Both groups of students initially described various tasks associated with the medical and nursing professions. Medical history and diagnostics as well as the collaboration with other actors are seen by both groups of students to be important duties within the respective other profession. After the conclusion of the joint course the nursing students added to the scope of duties of doctors by including, among other things, the treatment of patients (e.g. selection of a suitable therapy, an adjustment of the therapy in the course of the disease if necessary, documentation of treatment measures, etc.) (see Figure 2 (Fig. 2)). The medical students, on the other hand, when describing the tasks involved in nursing focussed in particular on the continual observation of patients and passing on patient information to the doctor (see Figure 2 (Fig. 2)).

##### 3.3.2. Appreciation

Inter-professional PBL influenced the mutual appreciation of both groups of students. Both prior to the commencement of the course and after its conclusion all medical students stated that they had “high” appreciation of the nursing profession. One nursing student was of this opinion before the interprofessional PBL began. After the conclusion of the interprofessional course four nursing students considered their appreciation of the medical students to be “high”.

##### 3.3.3. Interprofessional communication and collaboration

Even before the start of the interprofessional course both groups of students predominantly (n=9) rated their ability to engage in interprofessional communication and collaboration as “very good” and “good”. After the conclusion of the interprofessional PBL the nursing students stated that their ability with regard to both interprofessional communication and collaboration had improved. The medical students now evaluated their ability in both areas as predominantly “good”, having previously predominantly considered them to be “very good”.

#### 3.4. Implications for future implementation

##### 3.4.1. Particularly positive aspects of interprofessional PBLs and suggestions for improvement

Both groups of students indicated that they had particularly positive experiences with regard to

1. the composition and structure of the course,
2. the discussion of the content and the exchange among the students
3. the tutor.

**Figure 3:**
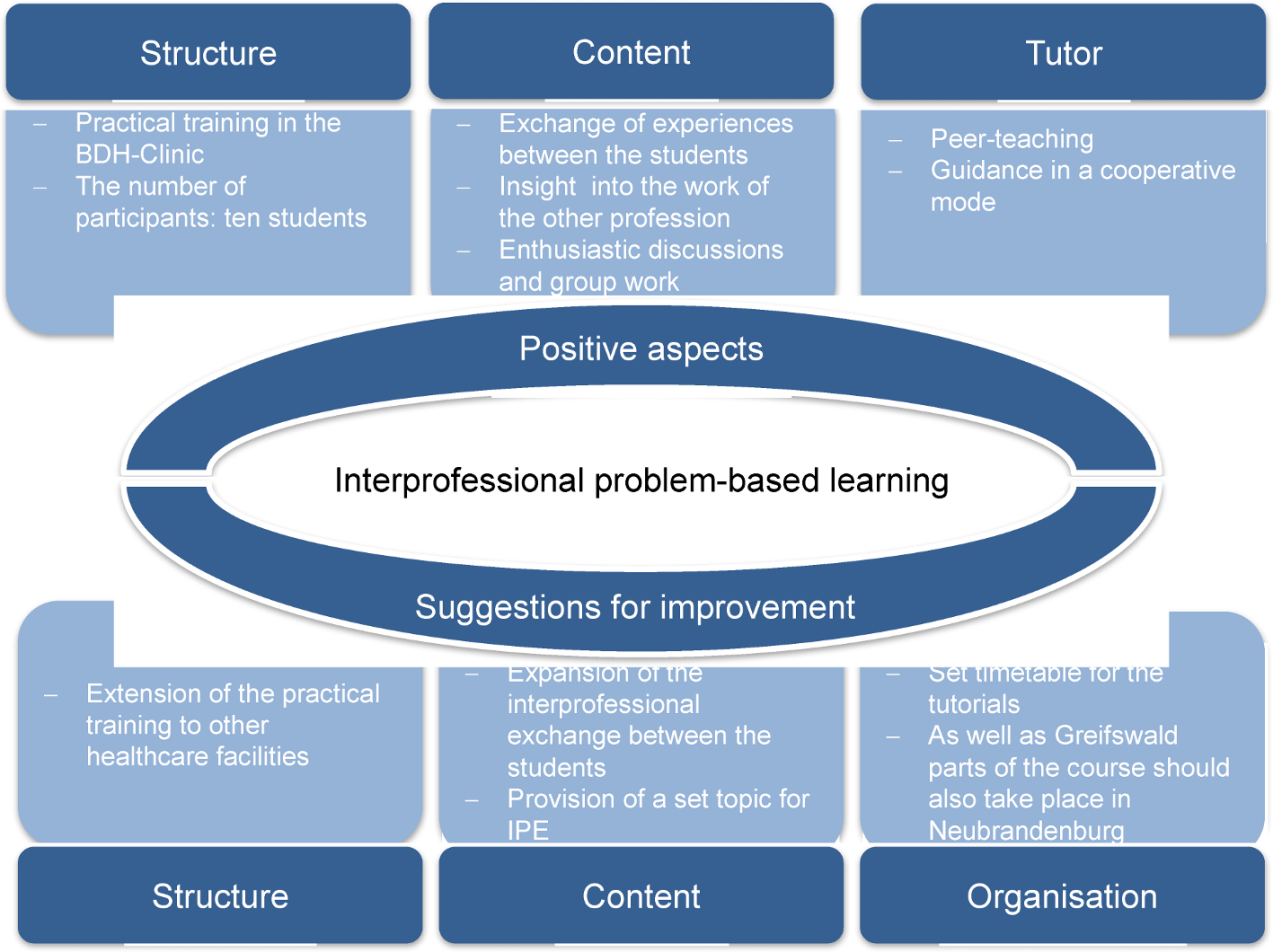
Positive aspects of the interprofessional PBL and suggestions for improvement (N=10)

The students emphasised that they found it positive that they were already able to have contact with patients at an early stage in their studies. This occurred in the context of the practical training in the BDH-Clinic (see Figure 3 (Fig. 3)).

Having gained an insight into the work of the respective other profession, both groups of students indicated that in future they would be better able to assess what the roles of the doctors and nurses are in the treatment of patients and where there are intersections in the collaboration.

For a sustainable implementation the students saw room for improvement in the planning and running of the interprofessional course (see Figure 3 (Fig. 3)). The students expressed that they would like the practical training to also take place in other healthcare facilities and the proportion of interprofessional exchange between the groups of students to be increased. In addition the students suggested that a stipulated topic for the assignment could improve the start of the group work and the active exchange. A set timetable for the tutorials and the course being conducted at both sites (UMG and HS NB) are, according to the students, further potential improvements to the interprofessional PBL.

##### 3.4.2. Identification of additional topics for interprofessional PBL

Both groups of students considered interprofessional PBL to be useful and after participating in the course were able to identify further topics which they deemed to be suitable for interprofessional PBL. The topics named can be classified as

**Figure 4:**
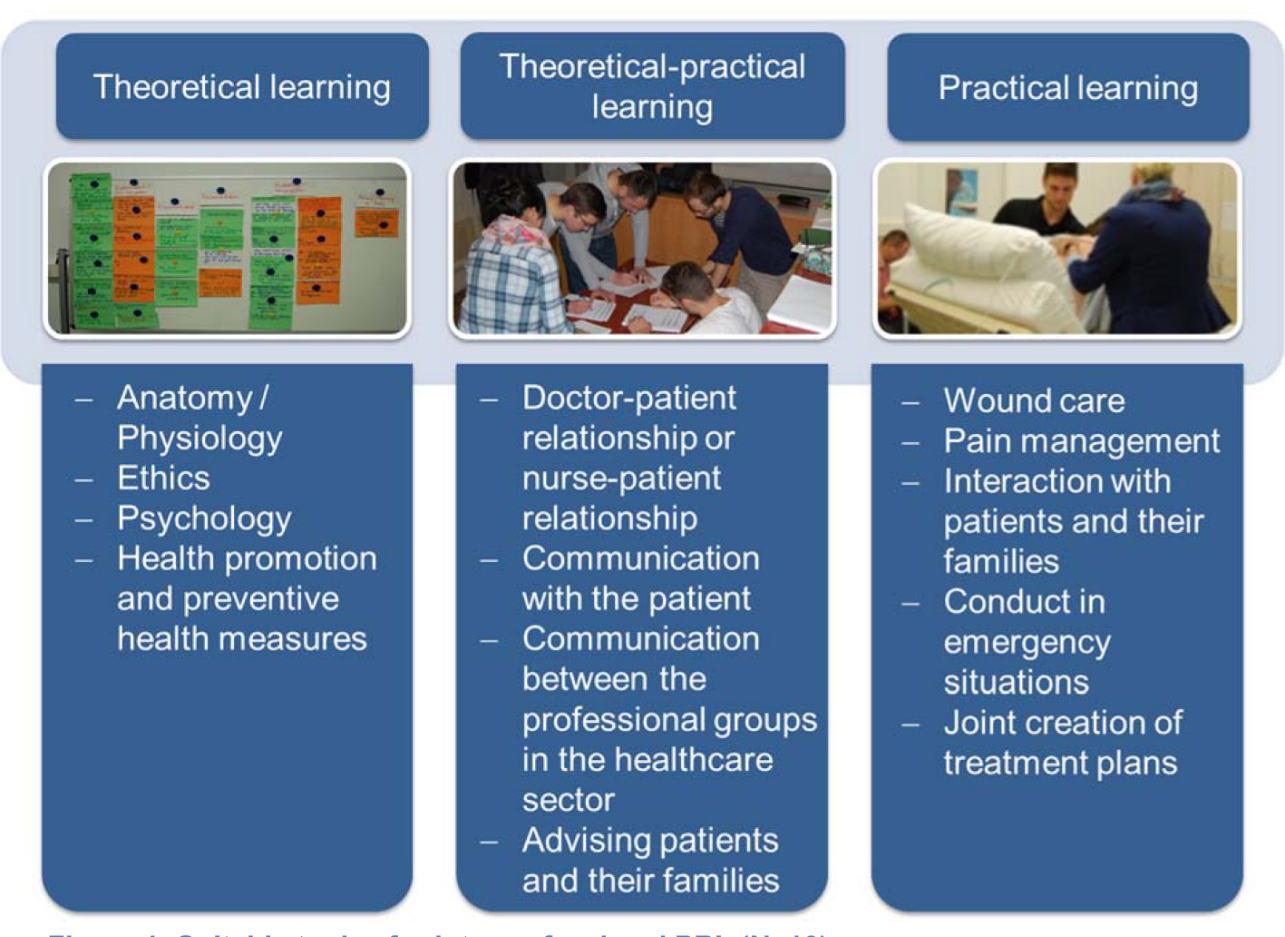
Suitable topics for interprofessional PBL (N=10)

1. theoretical (e.g. anatomy),
2. theoretical-practical (e.g. communication and advice) and also
3. practical teaching topics (e.g. pain management) (see Figure 4 (Fig. 4)).

## 4. Discussion

The students in the Bildungscluster study stated that they were able to expand their knowledge of the role of the respective other profession, that the interprofessional course had a positive impact on mutual respect and that the tutor had a positive influence on the interprofessional PBL through his cooperative learning guidance.

PBL challenges students to organise their own acquisition of knowledge and to intensify this knowledge through the exchanges in the group. This can lead to learners being dissatisfied with their learning outcomes and PBL causing frustration. Thus, the success of PBL is greatly influenced by the teaching skills of the tutor [17], [18], [19].

The students in the Bildungscluster study described what they viewed to be important roles of the medical and nursing professions. Interprofessional PBL should thus already occur early on during one’s training [20], in order to already convey fundamental knowledge about the roles at an early stage [21] and to solve tasks taking into account one’s own roles and those of the other occupational group [22]. PBL can thus enable students to flexibly perform tasks and roles in their day-to-day work in order to be able to appropriately care for patients [1], [23].

Solomon et al. and also Manek & Davidson outline how students learn to recognise the importance of the roles of individual occupational groups through interprofessional PBL and this then leads to improved appreciation [23], [24], [25]. More effective interprofessional teamwork and communication are the positive effects [2], [26], [27].

The students in the Bildungscluster study also reported that they assessed their ability to engage in interprofessional collaboration and communication to be either very good or good. The actual application in daily work would be the next necessary step. At the same time nine participants stated that they assessed themselves as having a high level of appreciation for the other profession. In the case of the four nursing students this was the case after the conclusion of the interprofessional PBL. Before the commencement of the interprofessional course the nursing students’ assessment of their level of appreciation of the medical students was “neutral”. In this context Areskog outlines the importance of leading students to have a positive attitude towards interprofessional collaboration at an early stage in their training. This occurs in his view by pointing out the positive effects of collaboration and leads to improved mutual understanding and trust [28].

After completion of the interprofessional PBL the students in the Bildungscluster study were of the opinion that they were able to actively work on the problem and the learning content in the course. In addition, they identified a number of other teaching topics which, in their view, could be dealt with using interprofessional PBL. These included anatomy/physiology, ethics and wound care.

Ethics [3], [29] and anatomy [30] are two topics which are also specified internationally in connection with interprofessional PBL. In addition, the following suitable topics are named: biochemistry, pathology, surgery and psychiatry [30], palliative care [3], [31], [32], back pain, depression [10] and HIV [11], [25]. However, to date, a systematic investigation of the suitability of topics for interprofessional PBL is unavailable. At the same time, the participants made suggestions for improvement in order to optimise the interprofessional PBL. In future the topic of the IPL will be defined in more detail, in order to make it easier for the group members to find the question to solve the problem. The aim is to thereby improve the link between theory and practice. 

The Bildungscluster study had its limitations. The first limitation pertains to the number of participants in the study. The Bildungscluster study Greifswald/ Neubrandenburg examined the implementation of interprofessional PBL with medical and nursing students. Therefore the course was first conducted with a group of ten participants in total. Internationally IPE pilot studies involving medicine and nursing in small groups (N=10 to 15 participants) are common [33], [34], [35]. The background is that the focus is on the development and initial implementation. Realising the course with a small number of participants has the advantage that the evaluation results for the first trial can be used to adapt IPE for a larger number of participants [36]. Nevertheless, with the small number of participants, one must assume that compared to other medical and nursing students those who became involved in the Bildungscluster study had a greater interest in IPE (selection bias). The results of the existing pilot study therefore cannot be fundamentally generalised.

The second limitation concerns the evaluation of the course. Internationally a number of valid instruments for the evaluation of IPE exist [37]. They include, among others, the “Readiness for Interprofessional Learning Scale” (RIPLS) and the University of the West of England Interprofessional Questionnaire (UWE-IP). A German version exists for both of these instruments. With regard to the RIPLS instrument Mahler et al. have come to the conclusion that there is need for further development due to the lack of factor stability [38]. Furthermore, RIPLS and UWE-IP primarily analyse the attitude of the participants towards IPL. The Bildungscluster study additionally examined the structure of the course, the guidance by the tutor and the identification of additional topics suitable for interprofessional PBL. For this reason a quantitative data collection instrument was developed specifically for the Bildungscluster study. The pre-test did not lead to any changes, which meant that the instrument developed was able to be used without any alterations.

## 5. Conclusions

The Bildungscluster study is one of the first studies in Germany to implement interprofessional PBL for medical and nursing students. The evaluation of the course was predominantly positive and in the view of the students it enabled learning under real-life care conditions. This result is in line with the findings from international studies. Nevertheless, Germany is at the beginning of the extensive implementation of interprofessional PBL. The first study results from the Bildungscluster study can be used for optimisation and in order to implement interprofessional PBL with a larger number of participants. Further studies are necessary to be able to comprehensively analyse the learning effects and the impact on everyday practice. Additionally, alongside the students, in the future instructors and persons responsible for everyday practice should also be involved in the evaluation.

Specific evaluation instruments are necessary for this purpose and should be developed in order to achieve comparability of study results. These are important prerequisites for interprofessional PBL to be implemented long-term in the regular curricula of medicine and nursing.

## Funding

This work was supported by the Stifterverband für die Deutsche Wissenschaft (Donors’ association for the promotion of humanities and sciences in Germany) under the grant number [H190 5907 9999 24586].

## Acknowledgements

The authors would like to thank the Stifterverband für die Deutsche Wissenschaft for making it possible to implement the Bildungscluster study Greifswald/Neubrandenburg. Special thanks go to the students who took part: the medical students from University Medicine Greifswald and the nursing students from the Department for Health, Nursing and Management at the University of Applied Sciences Neubrandenburg. Furthermore, we would like to thank all the instructors and the following people who were involved: Jens Thonack, Andreas Jülich, Sandra Huber, Ines Buchholz, Andreas Flick, Neeltje van den Berg, Nikolas Zimowksi, Angelika Beyer, Stefanie Kirschner. The Bildungscluster study was guided by a strategy group, whose commitment and enthusiasm contributed to the success of the study. We would therefore like to thank the members of the strategy group (in alphabetical order): Reiner Biffar, Jean-François Chenot, Wolfgang Gagzow, Peter Hingst, Anja Kistler, Arthur König, Christine Lorenz, Steffen Piechullek, Rainer Rettig, Hagen Rogalski, Helmut Schapper, Dirk Scheer, Sibylle Scriba, Elfi Thomas, Sven Wolfgram, Marek Tadeusz Zygmunt.

## Competing interests

The authors declare that they have no competing interests. 

## Figures and Tables

**Table 1:**
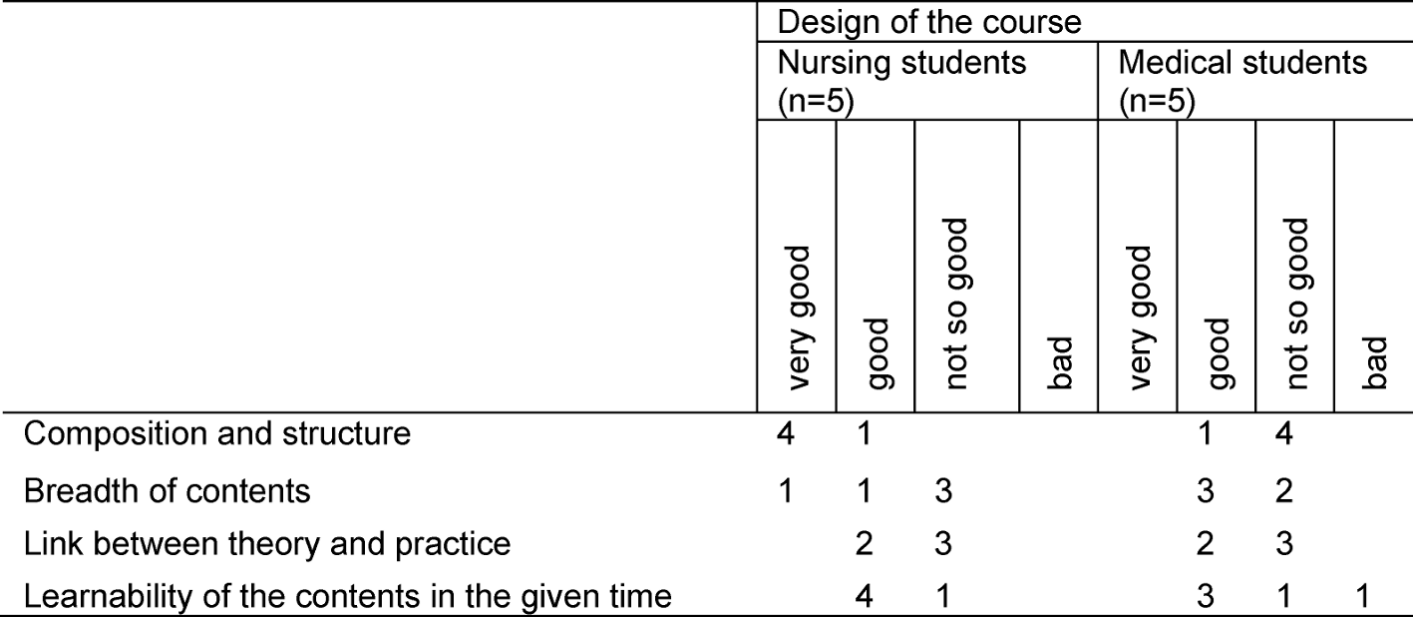
Evaluation of the design of the interprofessional PBL (N=10)

**Table 2:**
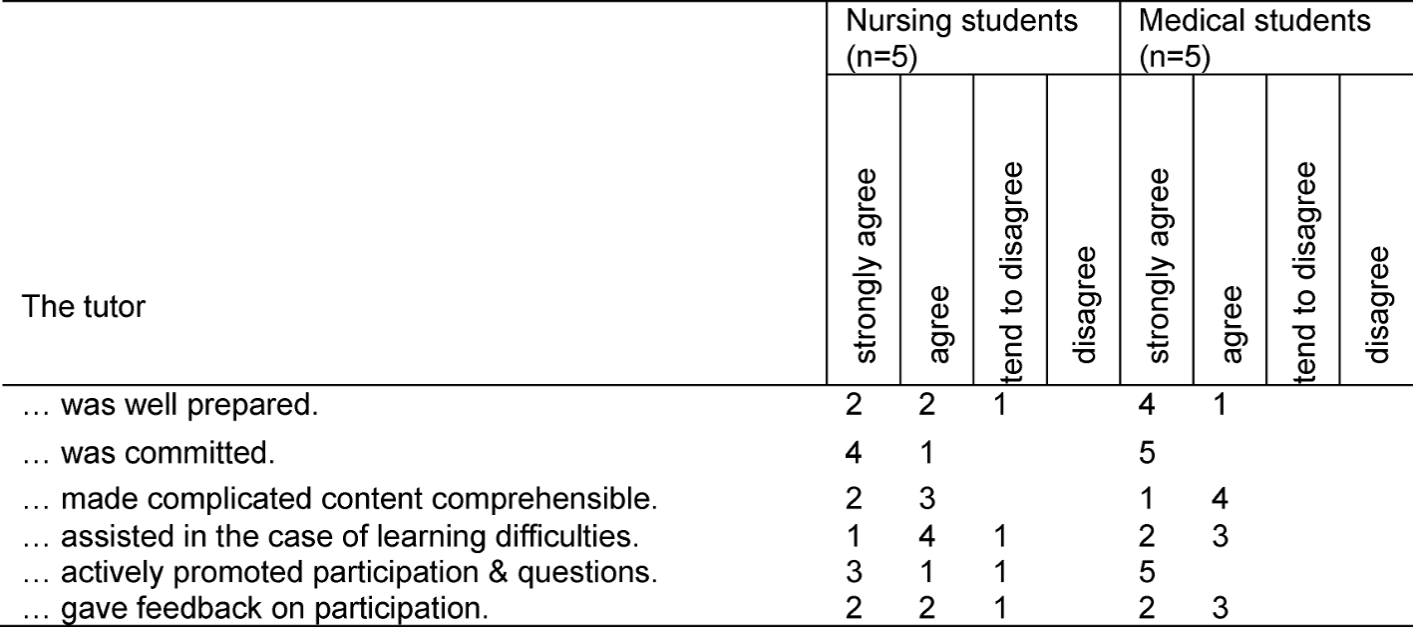
Evaluation of the tutor (N=10)
